# *Lemna minor* Cultivation for Treating Swine Manure and Providing Micronutrients for Animal Feed

**DOI:** 10.3390/plants10061124

**Published:** 2021-06-01

**Authors:** Reindert Devlamynck, Marcella Fernandes de Souza, Jan Leenknegt, Liesbeth Jacxsens, Mia Eeckhout, Erik Meers

**Affiliations:** 1 Provincial Research and Advice Centre for Agriculture and Horticulture (Inagro vzw), Ieperseweg 87, 8800 Roeselare, Belgium; jan.leenknegt@inagro.be; 2 Department of Green Chemistry and Technology, Ghent University, Coupure Links 653, 9000 Ghent, Belgium; Marcella.FernandesDeSouza@UGent.be (M.F.d.S.); erik.meers@ugent.be (E.M.); 3 Department of Food Technology, Safety and Health, Ghent University, Valentin Vaerwyckweg 1, 9000 Ghent, Belgium; liesbeth.jacxsens@ugent.be (L.J.); mia.eeckhout@ugent.be (M.E.)

**Keywords:** Lemnaceae, remediation, feed safety, mineral supplements, accumulation, agricultural wastewater, nutrient recovery

## Abstract

The potential of *Lemna minor* to valorise agricultural wastewater into a protein-rich feed component to meet the growing demand for animal feed protein and reduce the excess of nutrients in certain European regions was investigated. Three pilot-scale systems were monitored for nine weeks under outdoor conditions in Flanders. The systems were fed with a mixture of the liquid fraction and the biological effluent of a swine manure treatment system diluted with rainwater in order that the weekly N and P addition was equal to the N and P removal by the system. The design tested the accumulation of elements in a continuous recirculation system. Potassium, Cl, S, Ca, and Mg were abundantly available in the swine manure wastewaters and tended to accumulate, being a possible cause of concern for long-operating recirculation systems. The harvested duckweed was characterised for its mineral composition and protein content. In animal husbandry, trace elements are specifically added to animal feed as micronutrients and, thus, feedstuffs biofortified with essential trace elements can provide added value. Duckweed grown on the tested mixture of swine manure waste streams could be considered as a source of Mn, Zn, and Fe for swine feed, while it is not a source of Cu for swine feed. Moreover, it was observed that As, Cd, and Pb content were below the limits of the feed Directive 2002/32/EC in the duckweed grown on the tested medium. Overall, these results demonstrate that duckweed can effectively remove nutrients from agriculture wastewaters in a recirculated system while producing a feed source with a protein content of 35% DM.

## 1. Introduction

The rising world population and the improving living standards have been driving the increase of animal-based food consumption [1]. Global livestock production has been estimated to expand by 21% between 2010 and 2025 [2]. In this context, proteins play a pivotal role in animal feed as a source of nitrogen and essential amino acids [3]. Crop production, land-use change, processing, and transport contribute to the overall greenhouse gas emission of livestock production [4]. Therefore, there is an urgent need to develop resource-efficient and innovative practices to locally produce protein-rich feed alternatives with high areal productivity.

On top of that, European pig production is characterised by its intensiveness, which results in a manure abundance in certain regions [5,6]. Hence, the treatment and nutrient recovery of these waste streams have an essential role in improving the sustainability of conventional agriculture.

Duckweed has been shown to grow on wastewaters and subsequently produce a protein-rich feed ingredient suitable for pig production, offering a possible solution for addressing both the protein scarcity and local nutrient abundancy [7,8,9,10]. These small floating macrophytes occur all over the world and are the most rapidly growing Angiosperms, following a quasi-exponential growth rate [11]. Doubling times of 1.34 to 4.54 days are reported in optimal conditions [12]; however, its productivity depends on the climatic variation, the length of the growing season, and the management of the system. The estimated production rate in Europe is between 7 and 22 tonnes dry weight (DW) ha^−1^ yr^−1^ [13]. Besides productivity, duckweed’s key advantage is its high crude protein content of around 35% [14], and up to 45% DW [13]. In contrast to several major cultivated protein or starch crops, the entire plant is edible because of the lack of support tissues [15].

Although many studies have been conducted on the use of duckweed for the treatment of pig manure wastewaters, there are still several knowledge gaps. Most research has been carried out with either one large-scale pilot or replicates on a laboratory scale [10]. Phytoremediation aspects tend to focus on N, P, and biological oxygen demand (BOD); nevertheless, other elements are also present in wastewaters, which might gradually increase or decrease over time in a recirculated system. A gradual increase over time is considered as an accumulation, while a gradual decrease is considered as a depletion. Accumulation over time off, for example, Cl might eventually reach harmful levels and, hence, decrease the duckweed growth and environmental performance [16]. 

Besides duckweed cultivation, the produced biomass should also be suitable for animal consumption, and in this assessment, the mineral composition is generally overlooked. For example, Cu and Zn are frequently added to swine feed for improving feed efficiency [17,18], but these elements are only 10–20% absorbed by the animals. As a result, swine excrements have high concentrations of Cu and Zn [17]. Also, heavy metals like As, Cd, and Pb could have diverse toxicological health effects, including carcinogenesis, decreased reproductive ability, and damages to the nervous, skeletal, circulatory, endocrine, and immune systems of animals and humans [19]. Hence, these metals are regulated by European law for food and feedstuffs (respectively EC No 1881/2006 and Directive 2002/32/EC) [20]. 

To address these open questions, a pilot recirculation system was set up in triplicate at outdoor conditions and fed with a mixture of swine manure effluents. The elemental characterisation of the recirculated water and the produced duckweed was monitored over nine weeks to uncover trends in a continuous growing system and gather adequate data to compare with the mineral composition standards of animal feed. The aim was to identify if the elements K, Cl, S, Ca, Mg, Mn, Fe, Cu, Zn, As, Pb, and Cd would accumulate within a recirculated duckweed system using biological effluent and the liquid fraction of the swine manure treatment as nutrient sources. A second objective was to evaluate several mineral components (Mn, Cu, Fe, Zn, As, Cd, and Pb) in the duckweed biomass for potential nutritional and harmful effects in potential feed application. 

## 2. Materials and Methods

### 2.1. Experimental Set-Up

#### 2.1.1. Pilot

The growth was performed in 1000 L cubicontainers (BE COMPOSITE IBC, Mauser, Brühl, Germany), of which an area of 0.9 × 1.1 m was cut from the top. The sidewalls were covered with a black plastic foil to exclude light interference, which could trigger algae activity in the containers. A mesh (vidalXL, The Netherlands) with a pore size of 1.17 × 1.57 mm was placed over the cubicontainer to prevent that the insect *Cataclysta lemnata* would interfere with the experiment. On 22 August 2019, a pilot-scale growing experiment was inoculated with 500 g FW of *Lemna minor*. The identification of the duckweed species was performed using molecular barcoding based on plastidic markers prior to the experiment [21].

The experiment was conducted outdoors, and daily meteorological data, i.e., air temperature (°C), solar irradiance (W m^−2^), daylength (h), precipitation (mm m^−2^), and relative humidity (%), were received from the Belgian Royal Meteorological Institute for the complete growing season (Table A1). The solar irradiance was converted to light intensity, which is expressed in µmol m^−2^ s^−1^, using a conversion factor of 4.6 [22].

#### 2.1.2. Growing Medium

The starting medium was a mixture of rainwater, biological effluent (BE), and liquid fraction of pig manure (LF). These waste streams were sampled at the manure treatment facility of IVACO, Eernegem, Belgium. LF was obtained by separating the solid fraction from raw pig manure by centrifugation. This process reduces the P content in the LF [23]. Subsequently, the ammonia of the LF was nitrified and denitrified in separate tanks during the biological treatment, resulting in the BE stream with a reduced N content [23]. At Ivaco, the BE is partly further applied to arable land as a K-rich fertiliser, and partly treated to dischargeable water by a constructed wetland using reedbeds. 

BE and LF were sampled on 22 August 2019. 600 L of BE was stored in a closed cubicontainer (BE COMPOSITE IBC, Mauser, Brühl, Germany), while 50 L of LF was stored in a closed 50-litre feed barrel (PV-50L-HA) during the experiment. 

To determine the medium composition, a non-linear solver technique was performed using Microsoft Excel, and the results are given in Table 1. The aim was to maximise the LF composition within the following imposed constrictions:

1. the total mass of LF, BE, and RW equals 1000 kg, as this is the approximate limit of the cubicontainers,
2. all fractions of LF, BE, and RW are greater than zero,
3. the total N and total P content of the final mixture are below the limits proposed for *Lemna minor* [13],
4. the N/P ratio of the medium equals 3.0, as this is the ratio between the N removal and P removal determined in a duckweed system grown on diluted BE in outdoor systems [7].

Considering the concentrations of the three streams, data was gathered prior to the experiment as follows. First, it was assumed that rainwater (RW) would have a negligible N and P concentration. Furthermore, LF’s composition was extracted from a report on the valorisation of pig manure wastewaters in Flanders [24]. Finally, the concentration of the BE was extracted from an internal dataset containing T-N and T-P concentrations from the manure treatment facility that provided the LF and BE of this experiment. However, also samples were taken and frozen on the day of preparation and analysed afterwards to verify the theoretical composition.

**Figure 1:**
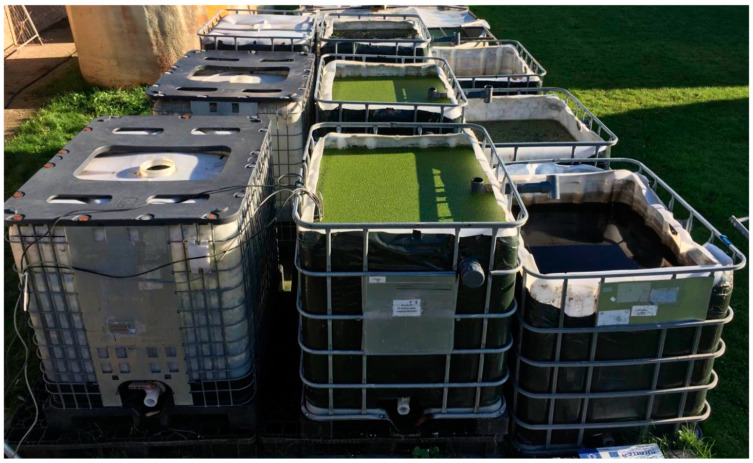
The three pilot-scale set-ups in which the influent is mixed and stored (**left**) before being pumped in the middle cubicontainer where duckweed is grown. With an overflow mechanism, the lower cubicontainer (**right**) is filled with effluent from the growing system.

First, the influent and cultivation cubicontainers were filled with the starting solution described in Table 1. During the growing period, the influent was transferred to the cultivation containers using a dosing pump (Etatron BT-MA/AD 50/3.0, Etatron, Italy) set at a debit of 800 L per week. Hence, via an overflow, the effluent container was filled (see Figure 1). 

The aim was to achieve a weekly loading rate of N and P that equalled the removal rate from the system. This was found to be 1.1 g N m^−2^ d^−1^ and 0.37 g P m^−2^ d^−1^ in duckweed cultivation systems on pig manure waste streams in Flanders [7]. Based on the concentrations mentioned in Table 1, BE and LF were added to the system in a ratio that allowed the added N and P to equal the removed. This resulted in the addition of 1 kg and 10 kg of respectively LF and BE. Furthermore, the influent cubicontainer was filled with the system effluent, achieving a recirculating system. 

During the process, the volume of each cubicontainer was determined by measuring the height of the water table. In this way, the mass flows throughout the system could be determined without neglecting the effect of evaporation and precipitation on the medium concentrations. 

#### 2.1.3. Sampling

Each week, duckweed was harvested and drip-dried for 5 min before weighing. Subsequently, 500 g of the harvested biomass was inoculated back in the cultivation cubicontainers, while the rest was oven-dried for three days at 60 °C to determine a representative DW percentage (DW%). This fully dried duckweed was used for dry weight, protein, and heavy metal determination. Furthermore, a water sample of 1 L was taken from each container and directly stored and −18 °C. For the influent, this was performed after filling the cubicontainer with the affluent, BE and LF. For the affluent, this was done prior to the addition of BE and LF. 

### 2.2. Plant Analysis

For the determination of the mineral composition (P, S, Ca, Mg, Na, K, Cu, Fe, Zn, As, Cd and Pb), an inductively coupled plasma optical emission spectrometry (ICP-OES, VISTA-MPX, Agilent Technologies, Santa Clara, CA, USA) was performed on the destructed plant samples. The dried plant samples were first homogenised using a Retch ZM-200 plant mill (Düsseldorf, Germany) with a 0.5 mm sieve. Subsequently, 0.25 g was weighted to digestion tubes containing Aqua regia (7.5 mL HCl with 2.5 mL HNO_3_) and 50 µL of a pure gold (Au) solution of 1000 mg L^−1^. Closed-vessel microwave destruction (MARS6, CEM, Matthews, NC, USA) was performed on these samples. Afterwards, the samples were diluted to 50 mL using Milli Q^®^ (Merck, Belgium).

Kjeldahl nitrogen (Kj-N) was measured according to Van Ranst et al., (1999) [25], without the addition of a reduction agent, using a distiller (Büchi auto Kjeldahl Unit K-370, Büchi, Switzerland), destructor (Büchi digest automat K438, Büchi, Switzerland), sampler (Büchi Kjeldahl sampler type K-371, Büchi, Switzerland) and scrubber (Büchi scrubber B414, Büchi, Switzerland). This method measures organic and ammonium nitrogen. Additionally, Kj-N content was used to calculate the protein content by multiplying it with the factor 6.25 [26]. 

The total N content (T-N) was determined according to the procedure of Dumas using a CNS analyser (Primacs SNC-100, Skalar, The Netherlands), as described in the guideline NEN- EN16168:2012 presented by the Royal Dutch Normalisation Institute (NEN). In this method, 200 mg of dried plant material is combusted, and the produced N_2_ is measured with a thermal conductivity sensor. As all nitrogen forms are combusted, this analysis gives the sum of organic, nitrate, nitrite, and ammonium nitrogen. With the T-N content, the N removal of the plant was calculated. 

### 2.3. Medium Analysis

For water analysis, an open vessel microwave destruction using a MARS6 (CEM, Matthews, NC, USA) was performed. A centrifuge tube was filled with 5 mL of the sample, 3 mL of 65% HNO_3_ (Chem-Lab, Belgium), and 3 mL of 30% H_2_O_2_ (Chem-lab, Belgium). For an optimal release, 50 µL of a 1000 mg L^−1^ Au solution was added. The mixture was subsequently destructed by a stepwise gradual increase of temperature to 100 °C. This temperature was held for 30 min before cooling to room temperature. Afterwards, the digested sample was diluted to 25 mL using Milli Q^®^ (Merck, Belgium). Subsequently, inductively coupled plasma optical emission spectrometry (ICP-OES, VISTA-MPX, Agilent Technologies, Santa Clara, CA, USA) was performed on the destructed samples for analysing P, S, Ca, Mg, Na, K, Cu, Fe, Zn, As, Cd, and Pb.

Chloride, NO_3_^−^, PO_4_^2−^, and SO_4_^2−^ were determined by liquid ion chromatography (850 Professional IC anion, Metrohm, Antwerpen, Belgium) in a 150 mm column (Metrosep A SUPP 5-150/4.0, Metrohm, Antwerpen, Belgium), following the ISO 10304-1:2007 method. 

Total N content (T-N) was determined according to the procedure of Dumas using a CNS analyser (Primacs SNC-100, Skalar, Breda, The Netherlands). 

Finally, pH and electric conductivity (EC) was measured with a pH-meter (ProfiLine pH 3110, WTW, Weilheim, Germany) and a conductivity tester, respectively (ProfiLine Cond 3110, WTW, Weilheim, Germany).

### 2.4. Calculations

First, biomass productivity or the linear growth rate (LGR) was calculated as follows [27]:

(1) $$\mathrm{LGR}\;=\frac{\left({\mathrm{DW}\%}_{\mathrm{end}}{*\mathrm{FW}}_{\mathrm{end}}-{\mathrm{DW}\%}_{\mathrm{start}}{*\mathrm{FW}}_{\mathrm{start}}\right)}{\mathrm{time}*\mathrm{surface}}\;\left[{g\;m}^{-2}{\;d}^{-1}\right]$$

Moreover, the N and P uptake by the plant and protein productivity were calculated by replacing the dry weight percentage (DW%) by, respectively, the T-N, T-P, and protein content and by replacing the fresh weight (FW) with the dry weight (DW) in Equation (1). 

The removal of an element by a duckweed system is weekly determined with Equation (2). Here, ‘t’ represents the conditions at the end of the week (before the addition of nutrients), and ‘t−1′ represents the conditions at the beginning of the week (after the addition of nutrients). Furthermore, the volume and concentration of the influent (V_I_ &V_I_), cultivation cubicontainer (V_C_ &V_C_), and effluent (V_E_ &V_E_) are used for each specific parameter. This is subsequently divided by the surface area of the system (0.99 m^2^) and duration between two measurements (7 days).

(2) $${\mathrm{Removal}}_{t}=\frac{{\left(V_{I}{*C}_{I}+V_{c}{*C}_{c}+V_{E}{*C}_{E}\right)}_{t}-{\left(V_{I}{*C}_{I}+V_{c}{*C}_{c}+V_{E}{*C}_{E}\right)}_{t-1}}{{\mathrm{surface}*\mathrm{duration}}_{\mathrm{week}}}\;[\mathrm{mg}\;m^{-2}\;d^{-1}]$$

From the removed nutrients, only a part is taken up by the duckweed. The others remain in the growing medium or are removed by processes like sedimentation of particles containing nutrients or by conversion into gaseous forms like in volatilisation or denitrification. The contribution of duckweed in the nutrient removal is indicated with the relative uptake (%) and is calculated by dividing the nutrient uptake by the removal of the nutrient [28]. 

Although elements are removed in a duckweed system, each week nutrients are added by adding 10 kg BE and 1 kg LF. Therefore, the net balance between addition and removal can still be positive or negative, leading to a gradual increase or decrease over time. This phenomenon is respectively defined as an accumulation or depletion. The average daily increase or decrease of an element will be determined by linear regression using R. The slope of the regression is equal to the daily concentration increase. This is also defined as the accumulation rate in this manuscript. The significance of the increase or decrease over time was tested with a Pearson correlation function between the parameter and the duration of the experiment. The significance was evaluated on a 5% significance level (*p* < 0.05). Furthermore, the accumulation rate depends on both the plant performance as the system input. Therefore, this parameter only shows the gradual build-up or depletion of a respective element in the tested system. Finally, a negative result should be interpreted as depletion of a particular element in the system.

## 3. Results and Discussion

### 3.1. Medium Composition and Accumulation of Elements

During the experiment, the composition of the growing medium and the accumulation of elements was monitored. These conditions are summarised in Table 2, along with the optimal and maximal growing ranges for duckweed found in the literature [13,29]. When a certain component is within the optimal range, the growth will be optimal; when it is outside the maximal growing range, duckweed growth is theoretically impossible. In all other cases, duckweed growth is suboptimal. 

Comparing the optimal and maximal growing ranges with the composition of the growing medium shows that the medium is suboptimal. EC, pH, T-N, K, Cl, Mg, and Fe are in optimal ranges, but a reduction of T-P and an increase of S and Ca theoretically improve the productivity. 

It should be noted that the design of the experiment led to an optimal N and P content in the growing medium. These concentrations exceeded the discharge levels in Flanders for a manure treatment facility of 15 mg N L^−1^ and 2 mg P L^−1^ [30]. This effluent could be land applied or further treated to meet the discharge criteria. Treatment can be performed by the principle of a constructed wetland where several lagunes are placed in series. There is a various set of plants which can grow in the lagunes and remove the nutrients from the water. The processes in the system result in nutrient depletion, making the wastewater dischargeable without risk for eutrophication. However, it should be taken into account that the lower the nutrient content, also the lower the phytoremediation potential, and the protein composition of duckweed will be [7,28,31].

**Figure A1:**
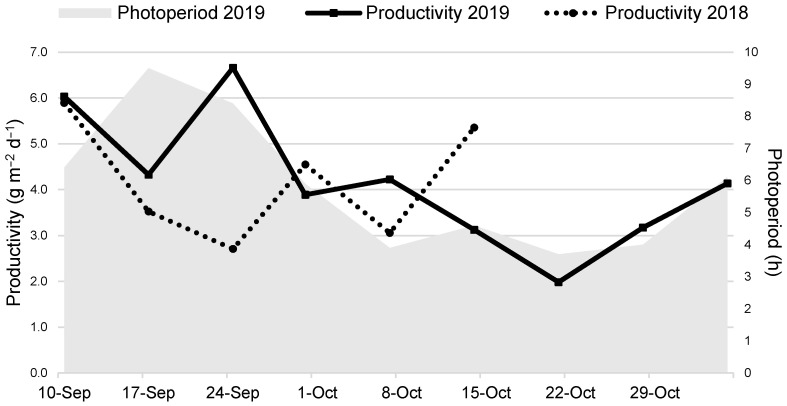
Duckweed productivity and the average daily photoperiod during the experiment compared with the productivity reached in a similar experiment using 22% of biological effluent from a pig manure treatment facility in 2018 [7].

The average productivity of the period from 28/08 until 4/11 was 4.5 ± 1.7 g m^−2^ d^−1^, and productivity peaked at the harvest of 24/09 with 6.6 ± 0.3 g m^−2^ d^−1^. In that week, there was an average temperature of 15 °C, an average light intensity of 99 ± 15 µmol m^−2^ s^−1^, and an average photoperiod of 8.34 h. The average productivity was lower than the one found in a previous study of duckweed grown on a diluted biological effluent from the same pig manure treatment facility, which resulted in an average productivity of 6.1 g m^−2^ d^−1^ and 10.7 t ha^−1^ yr^−1^ [7]. The latter was, however, reached in a full growing season including summer, which is a generally more productive season. The maximum duckweed growth rate in that study was 9.7 g m^−2^ d^−1^ at a weekly average temperature of 21 °C and light intensity of 115 µmol m^−2^ s^−1^ for 13.2 h [7]. A comparison of both studies at the same time period shows that current results are respective for its growing period (Figure A1). These results indicate that production levels would be higher if produced for a full growing season. An optimal temperature for duckweed growth depends on the species and ranges between 20 and 31 °C [32]. An optimal photoperiod at a light intensity of 371 µmol m^−2^ s^−1^, amounts 12–13 h [32]. Therefore, it is concluded that, besides some suboptimal concentrations in the growing medium, temperature, light intensity, and photoperiod were suboptimal.

**Figure 2:**
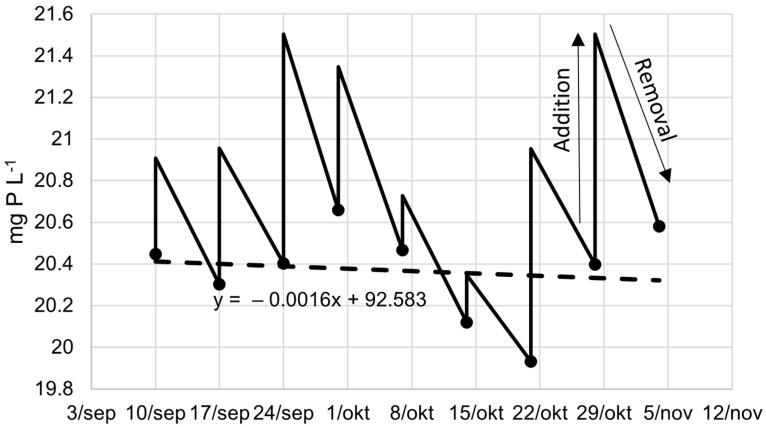

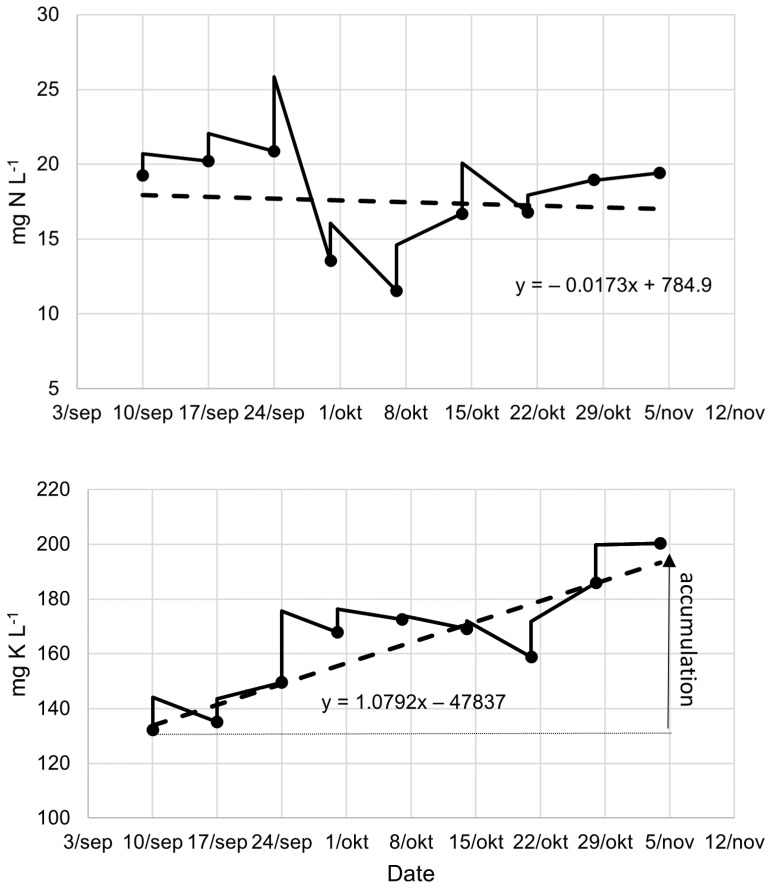
Average (**top**) total phosphorus, (**middle**) total nitrogen, and (**below**) potassium content in the growing media of the three cultivation systems (full line) with the linear regression of the concentration over time (dashed line).

In Figure 2, the compositions show a sawtooth pattern because of the weekly addition of LF and BE, which contain nutrients that are removed afterwards by the combination of duckweed uptake and other removal mechanisms like sedimentation or denitrification. In the figure of total P and total N, the removal of nutrients is approximately equal to the addition however, variation occurs. This variation can be addressed to sampling, laboratory handling, and precipitation and evaporation in the system. In the figure of potassium, the removal by the system is smaller than the weekly addition (Figure 2). Hence, the K concentration in the growing medium increases gradually. Within this manuscript, this is defined as accumulation. If the concentration gradually decreases, this will be defined as depletion. In the case of T-N and T-P, it could be argued that there is a small depletion however, there was no significant correlation with the duration over time, meaning that the concentration was constant. In contrast, the correlation of K with the duration of the experiment was significant, meaning that the accumulation of K was significant (*p* < 0.05). 

The accumulation rate of K can be estimated by the slope of the regression line, which is 1.0792 mg K L^−1^ d^−1^. And indeed, after the experiment of 55 days, the K concentration should increase 55 times 1.0792 mg L^−1^ or 59.36 mg L^−1^. The starting concentration was 132 ± 9, and the end concentration was 200 ± 13 (Table 2). The difference between start and end is, however, not exact 59.36 mg K L^−1^ but the figure also shows that the final concentration is slightly above the regression line (Figure 2). The correlation factor is a good way to estimate the average accumulation rate. This discussion can be done for each element and is summarised in Table 3.

As the correlation of the T-N and T-P was not significant (Table 3), the goal of adding as much N and P to the system as it could remove was achieved, indicating that the non-linear solver technique predicted effectively the weekly required amount of LF and BE. Furthermore, analyses showed that BE and LF had an N content of respectively 301 ± 42 and 4665 ± 756 mg N L^−1^ and a P content of respectively 60 ± 3 and 206 ± 61 mg P L^−1^. The observed compositions are in line with the average values found for LF and BE in Flanders [24]. Nevertheless, the starting conditions were not correctly predicted with the solver technique. The T-P content was twice as high as the result from the solver technique (20 instead of 10 mg L^−1^), while the actual T-N of the starting condition was lower (20 instead of 33) (Table 1 and Table 2). When preparing the growing medium, the BE is taken from a valve positioned at the bottom of the cubicontainer. Presumably, there is a sedimentation layer in the cubicontainer vessel of BE. The fraction of sediment was higher when preparing the growing medium. The solid fraction of pig manure contains generally a higher P and lower N content than the liquid fraction [33], which can explain the observed deviations.

Contrary to N and P, other elements tended to increase in the continuous cultivation (Table 2 and Table 3). With the accumulation rates, a first extrapolation can be performed, although the removal rates might variate over different growing periods, and a determination over a full growing season and different years would give a more precise determination of the yearly accumulation rate. Electric conductivity, nitrate, K, Cl, T-S, Ca, and Mg rose steadily and would not have any impact on the growth performance of duckweed after one growing season of 175 days, which can be considered a feasible length of the growing season in Flanders [7]. For example, in the studied conditions, Cl content significantly increased, which might eventually induce salt stress. With the monitored accumulation rate of 0.18 mg L^−1^ d^−1^, a discharge limit of 1000 mg L^−1^ would only be reached after 5466 days or 31 growing seasons of 175 days. This limit was imposed on pig manure treatment facilities in Flanders with a capacity of more than 60,000 tons of manure per year [30]. Even then, N and P removal by duckweed might not be affected, as this is only significantly reduced at a Cl concentration of 1772.5 ppm [34]. This should be a point of attention, as the chemical oxygen demand (COD) tends to increase at high salt concentrations [34]. Another important element to monitor is K, which had the highest accumulation rate in the system and would exceed the maximal growing range (2000 mg K L^−1^) in 1713 days or 9.8 equivalent growing seasons. 

In contrast, the differences in the concentration of Fe, Zn, and Cu were small to be statistically significant, while Mn showed a depletion over time. It was not possible, however, to draw a conclusion for potentially harmful elements like As, Cd, and Pb because all values found were below the limit of detection (LOD). These results indicate that more focus should be given to the accumulation effects on continuous recirculation cultivation at a large scale to determine at which point the water should be discharged, and the system should be restarted without losing productivity or treatment capacity.

### 3.2. Mineral Removal and Recovery Potential 

Removal in a duckweed system is a combination of nutrient uptake by the plant and sedimentation. Except for carbon and nitrogen, which can also be removed in gaseous form by biodegradation [17]. The removal by the duckweed system and the contribution of the plant uptake are summarised in Table 4. For most of the monitored elements, duckweed’s uptake was much lower than the system’s removal, indicating that phytoremediation was only partly responsible for the wastewater treatment obtained.

The relative N uptake in this study was 24%, which is similar to the relative N uptake of 28% found in a duckweed pond fed with swine manure wastewater with a high nutrient load [28].

Remarkably, all K that was removed was taken up by the plant, which indicates that none was sedimented. Potassium is a more exchangeable element in wetland soils, making it highly mobile and explaining the observed result [35]. All removed K would re-enter the livestock system in the hypothesis that duckweed is used as a feed source. 

The N and P removal are comparable to other duckweed treatment systems. Zimmo et al. found an N and P removal range of respectively 1.09–1.36 g N m^−2^ d^−1^ and 0.104–0.154 g P m^−2^ d^−1^ [36]. The N removal rate is comparable to a study on a reed-based constructed wetland which was monitored on a large scale in a temperate maritime climate, observing an N removal in summer of 1.22 g m^−2^ d^−1^ and 0.75 g m^−2^ d^−1^ in autumn [37].

With the removal rates found in the present study and considering a growing season of 175 days [7], it was extrapolated that 255 m^3^ LF and 2550 m^3^ BE per growing season would have been added and treated in a 1-ha duckweed pond with similar conditions to the experiment. The calculated treatment capacity was linked to the size of the farm and its pig places. A pig place is the average count of pigs present at the farm, taking into account that there are several production cycles in a year and that there are periods where no pigs are housed between two cycles. The average manure production was approximately 1.2 ton per year per pig place present at a farm in the period between 1998 and 2007 [38]. Furthermore, 0.83 m^3^ of BE and 0.87 m^3^ of LF are produced from 1 m^3^ of raw manure with a density of 0.99-ton m^−3^ [23]. It should be noted that the extrapolation towards a hectare scale is a big step from the tested conditions. Therefore, an up-scaling validation is required to determine a more precise value. The manure generated by a farm with a housing capacity of 2805 pigs can be treated in the proposed duckweed system of 1 ha if the raw manure is pre-treated with a separation step followed by a biological treatment with an average performance. Hence, a considerable share could be treated of the manure produced in the manure facility which provided the wastewater, as this facility had a housing capacity of approximately 3000 pigs.

In this study, the swine wastewater was added to the pond after centrifugation and biological treatment (BE and LF) because this is a widespread treatment in Flanders [5]. Un-treated or anaerobically digested wastewater can also be treated using duckweed [9,10]. The N/K ratio will be different in these waste streams. As a result, the accumulation rate of K in a continuous recirculated system will be smaller. The same process holds for other accumulated elements. The downside is, however, that the areal need for processing the wastewater will be larger as these pretreatment techniques have a smaller reduction effect on the N and P concentration of the wastewater. 

Finally, the removal rates might help to identify the potential accumulating elements for any given wastewater. Although, caution and validation over a full growing season at a large scale are advised, as the environmental performance depends on the growing medium and climate.

### 3.3. Feeding Value 

**Figure A2:**
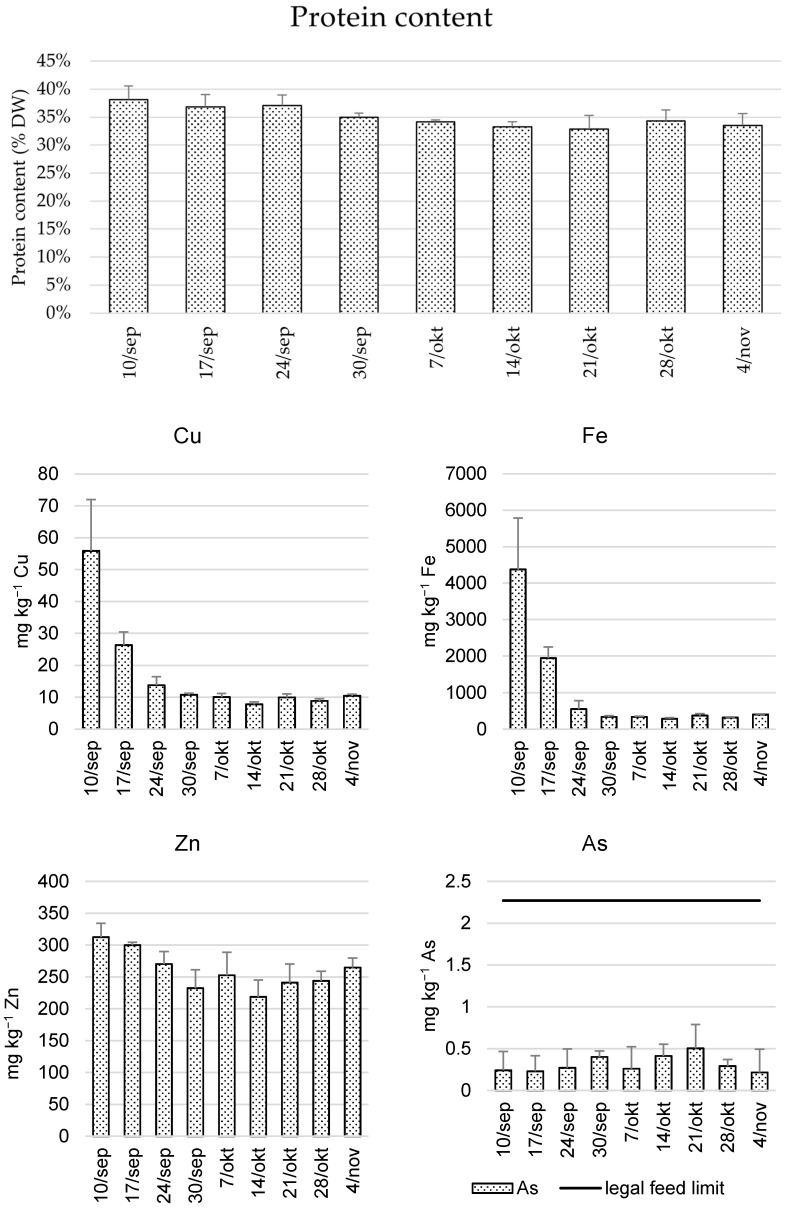

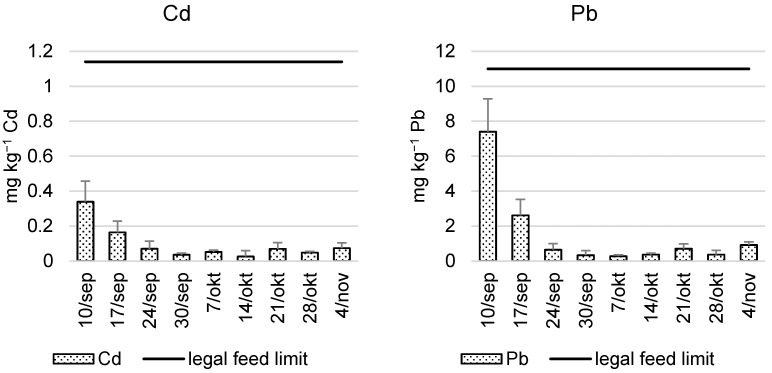
Protein, Fe, Zn, Cu, As, Cd, and Pb concentration with standard deviations (error flag) of the three duckweed Scheme 2019. *7*, 221–243, doi:10.1146/annurev-animal-030117-014838.

After harvesting, duckweed could be used as a feed ingredient. This can be done fresh after harvest, or when it is dried, or when proteins are extracted. An ingredient is considered protein-rich by the EU protein balance sheet when the protein content is between 30–50 g per 100 g product [39]. When fully dried, duckweed contained 35 ± 2% DW and peaked at 10/09 with a protein content of 38 ± 2% DW (Figure A2). This is in line with the study of Zhao et al. [14]. This protein content allows classifying the produced duckweed as protein-rich biomass. 

However, this holds for a fully dried product. The more moisture a product has, the more the contained elements are diluted. Duckweed should be almost fully dried before being considered a protein-rich product. The moisture content influences the mineral composition of a product. Therefore, all units are converted to a fully dried product in this manuscript to enable comparison with legal or suggested limits. 

Copper, Fe, Zn, and Mn are known to have an essential role in metal-containing enzymes and lead to an improved immunity of livestock [40]. Conventional feeds are deficient in microelements, and for this reason, there is a need to supplement these constituents to livestock natural feeds [41]. This is also visible in the data presented in Table 5. Commodities like corn, oat, wheat, and steamed potatoes have Mn, Zn, and Fe levels below the feeding standard of both laying hens and swine (Table 5). No conventional feed ingredient can provide Zn levels that are close to 150 mg kg^−1^ DM. Duckweed grown in this study, however, has a much higher Zn, Mn, and Fe concentration than other commodities, while it has similar Cu values. It can be considered as a source of Mn, Zn, and Fe, and adding duckweed in the feed could act as a replacer for these elements in livestock feed. Nevertheless, duckweed cannot be unlimitedly mixed in feed, as its Zn content surpasses the proposed maximum of 150 mg Zn kg^−1^ DM in complete feed for piglets and sows [42].

Additionally, the elements As, Cd, and Pb in feedstuffs are regulated by Directive 2002/32/EC of the European Parliament [20] and may not exceed the respective limits of 2, 1, and 10 mg kg^−1^ feedstuff. It should be noted that these limits do not hold for a completely dried product but a feedstuff with a water content of 12%. Converting the limits to the unit mg kg^−1^ dry weight results in the respective thresholds of 2.3, 1.1, and 11 mg kg^−1^ dry weight. These limits were not exceeded in the cultivation period between 10/09 and 4/11. Hence, duckweed can safely be used as a feed source when cultivated accordingly to this study, concerning As, Cd, and Pb (see Table 4 and Figure S2). It should be noted that there are several more aspects that should be monitored to guarantee feed safety, such as pathogens, viruses, and xenobiotics [19].

For all micronutrients analysed (Fe, Zn, Cu, As, Cd, and Pb), the contents do not seem to accumulate in the plant over time (see Figure S2). In contrast, the compositions sharply drop for Fe, Cu, Cd, and Pb, within the first three weeks, stabilizing afterwards. The comparison of the Fe and Zn concentrations with the suggested levels is complex. Therefore, it was chosen to compare the only concentrations from the stable period, being 30/09 to 4/11. This was not preferred for Cd and Pb, as safety should always be guaranteed. 

The decreasing trend might suggest a reaction of the duckweed to the depletion of these nutrients within the growing medium. However, the Fe content in the medium does not sharply drop over time (Table 3). Arsenic, Cd, and Pb were all under the detection limit, suggesting nor a high content at the start nor the end of the experiment. Therefore, it is suggested that the high heavy metal concentrations of duckweed at the beginning of the experiment are caused by a historical accumulation at the sourcing location, which is subsequently thinned out by the continuous cultivation and harvest. 

The observed high initial content is however, disadvantageous as the starting concentration of Pb is very high and close to the legal feed limit. Caution is advised at the start of the cultivation, and inoculating duckweed with As, Cd, and Pd concentrations that match the legal limits would minimise the risk in the first weeks. 

## 4. Conclusions

With the help of a non-linear solver technique, a combination of liquid fraction and biological effluent from a swine manure treatment facility was treated with duckweed. The systems were fed with a mixture of the liquid fraction and the biological effluent of a swine manure treatment system diluted with rainwater in order that the weekly N and P addition was equal to the N and P removal by the system. The N and P concentrations in the system were high in order to have optimal duckweed growth. Potassium, Cl, S, Ca, and Mg showed an accumulation tendency within the wastewater. In a continuous recirculation growing system, these elements can eventually cause stress for duckweed cultivation. Nevertheless, N and P were removed from the growing medium, and protein-rich biomass was produced, with a content of 35 ± 2% dry weight. The mineral composition was rich in Mn, Zn, and Fe and can be seen as a source of these elements. The potential harmful heavy metals (As, Cd, and Pb) were monitored and were below the feed limits proposed by Directive 2002/32/EC. Hence, duckweed has the potential to be used to treat swine manure wastewater while producing a mineral- and protein-rich feed ingredient. 

## Figures and Tables

**Table 1:** The total N, total P and the N to P ratio of LF, BE, and RW (with LF = liquid fraction raw swine manure, BE = Biological effluent of a pig manure treatment facility, and RW = rainwater), together with the calculated composition of the mixture after a non-linear solver technique maximising the LF composition within presented restrictions. The restrictions were found in literature [7,13].

	Total N	Total P	Ratio	Mass
	[mg kg^−1^]	[mg/mg]		[kg]
LF	4800	225		4
BE	331	247		41
RW	0	0		955
Mixture	33	11	3.0	1000
Restrictions	350	11	3.0	

**Table 2:** Average composition ± standard deviations of the cultivation cubicontainers at the start and end of the experiment; these compositions are compared with optimal and maximal growing ranges (where LOD means Limit of Detection).

	Start (10 September 2019)	End (28 October 2019)	Optimal Growing Ranges	Maximal Growing Ranges	Unit
pH	7.0 ± 0.1	6.8 ± 0.1	6.5–7.5 ^α^	5.0–9.0 ^α^	
EC	1.0 ± 0.1	1.1 ± 0.1	0.6–1.4 ^α^	<10.9 ^α^	mS/cm
T-N	19 ± 2	19 ± 1	2.8–350 ^α^	<2100 ^α^	mg L^−1^
NO_3_^-^	0.2 ± 0.2	5.6 ± 0.3	2.8–350 ^α^	<2100 ^α^	mg L^−1^
PO_4_^3-^	0.8 ± 0.0	0.5 ± 0	0.4–11 ^α^	<55 ^α^	mg L^−1^
T-P	20 ± 0	21 ± 0	0.4–11 ^α^	<55 ^α^	mg L^−1^
K	132 ± 9	200 ± 13	39–780 ^α^	<2000 ^α^	mg L^−1^
Cl	16 ± 3	29 ± 3	0.4–36 ^α^	<3500 ^α^	mg L^−1^
T-S	17 ± 1	23 ± 2	48–1900 ^α^	<4800 ^α^	mg L^−1^
Ca	15 ± 0	17 ± 0	20–400 ^α^	<2000 ^α^	mg L^−1^
Mg	5.9 ± 0.3	8.8 ± 0.7	5.0–97 ^α^	<1200 ^α^	mg L^−1^
Fe	0.53 ± 0.07	0.51 ± 0.06	<27.9 ^β^	<100 ^β^	mg L^−1^
Mn	16 ± 14	9 ± 7	<54,900 ^β^	<274,500 ^β^	µg L^−1^
Cu	8.3 ± 6.3	3.4 ± 2.6	<3200 ^β^	<6300 ^β^	µg L^−1^
Zn	28 ± 23	11 ± 8	<6500 ^β^	<65,300 ^β^	µg L^−1^
As	<LOD	<LOD			
Cd	<LOD	<LOD			
Pd	<LOD	<LOD			

Source: ^α^ [13], ^β^ [29].

**Table 3:** Summary of the daily accumulation rate of several parameters with their according significance value and interpretation.

Parameter	Accumulation Rate	Unit	*p*-Value	Interpretation
pH			0.432	Constant
EC	3.88	mS cm^−1^ d^−1^	0.042	Accumulation
T-N			0.541	Constant
NO_3_^−^	0.12	mg L^−1^ d^−1^	0.000	Accumulation
PO_4_^3−^	−0.004	mg L^−1^ d^−1^	0.000	Depletion
T-P			0.685	Constant
K	1.09	mg L^−1^ d^−1^	0.000	Accumulation
Cl	0.18	mg L^−1^ d^−1^	0.000	Accumulation
T-S	0.11	mg L^−1^ d^−1^	0.000	Accumulation
Ca	0.037	mg L^−1^ d^−1^	0.0004	Accumulation
Mg	0.045	mg L^−1^ d^−1^	0.000	Accumulation
Fe			0.363	Constant
Mn	−0.15	µg L^−1^ d^−1^	0.022	Depletion
Cu			0.402	Constant
Zn			0.662	Constant
As	<LOD			<LOD
Cd	<LOD			<LOD
Pd	<LOD			<LOD

**Table 4:** Average composition, uptake rate, removal rate and relative uptake rate of the removal by duckweed ± standard deviations (LOD stands for limit of detection).

	Content	Uptake	Removal	Relative Uptake
	[mg g^−1^ DW]	[mg m^−2^ d^−1^]	[mg m^−2^ d^−1^]	
N	60 ± 4	264 ± 123	1107 ± 715	24%
P	13 ± 1	58 ± 31	149 ± 150	39%
K	52 ± 10	233 ± 85	72 ± 3002	102%
S	6.1 ± 0.8	27 ± 15	47 ± 318	59%
Mg	2.8 ± 0.2	12 ± 5	32 ± 103	38%
Ca	11 ± 1	44 ± 18	242 ± 383	18%
Fe	1 ± 1	4.7 ± 14.5	44 ± 63	11%
Zn	0.25 ± 0.04	1.2 ± 0.7	2.4 ± 2.8	49%
	[µg g^−1^ DW]	[µg m^−2^ d^−1^]	[µg m^−2^ d^−1^]	
Cu	16 ± 15	82 ± 179	910 ± 1100	9%
As	0.32 ± 0.19	1.2 ± 2.0	<LOD	
Cd	0.1 ± 0.1	0.43 ± 1.05	<LOD	
Pb	3.0 ± 5.2	0.41 ± 11.36	<LOD	

**Table 5:** Recommended levels of micronutrients in livestock feed and the content of these elements in conventional feeds per kg dry matter (DM), adapted from Chojnacka (2008) [41], and duckweed (results from the present study ± standard deviations). The data of soybean meal was retrieved from Feedipedia [43].

Feeding Standard/Material	Mn	Zn	Cu	Fe
	[mg kg^−1^ DM]	[mg kg^−1^ DM]	[mg kg^−1^ DM]	[mg kg^−1^ DM]
Feeding standard for laying hens	60–70	50–60	5–6	60–70
Feeding standard for swine	30–40	20–165	70–150	90–100
Maximum limit piglets & sows		<150		
Corn (grain)	5	15	3.3	26
Oat (grain)	38	18	3.3	52
Wheat (grain)	26	23	2.7	43
Rye (grain)	58	26	3.3	60
Potatoes (steamed)	3	5	1.1	16
Fodder yeasts	14	9	12.6	90
Soybean meal	44	57	17	201
Duckweed	410 ± 60	250 ± 40	10 ± 2	372 ± 87

## Data Availability

Data is contained within the article and supplementary material.

## Supplementary Materials

The following are available online at https://www.mdpi.com/article/10.3390/plants10061124/s1, Figure S1: Comparing productivity, Figure S2: Protein.

## Appendix A

**Table A1:** Weather data.

Date	Average Temperature (°C)	Average Relative humidity (%)	Average Light Intensity (µmol m^−2^ s^−1^)	Average Photoperiod (h)		Total Precipitation (mm)
10/Sep	13 ± 2	82 ± 5	112 ± 14	6.4 ± 1.9		18.9
17/Sep	16 ± 2	83 ± 8	101 ± 16	9.5 ± 2.7		0.7
24/Sep	15 ± 0	72 ± 3	99 ± 15	8.4 ± 2.5		5.7
30/Sep	15 ± 2	91 ± 6	106 ± 20	5.9 ± 1.4		44
7/Oct	13 ± 2	86 ± 4	155 ± 64	3.9 ± 1.9		49.7
14/Oct	14 ± 2	89 ± 6	114 ± 36	4.6 ± 3.0		17.5
21/Oct	13 ± 2	90 ± 5	135 ± 69	3.7 ± 2.4		21.1
28/Oct	13 ± 3	90 ± 6	92 ± 30	4.0 ± 2.6		9.4
4/Nov	9 ± 3	90 ± 4	74 ± 9	6.1 ± 1.4		23.3
Average	14 ± 4	83 ± 9	109 ± 36	7.0 ± 3.5	Total	190.3
